# Federated benchmarking of medical artificial intelligence with MedPerf

**DOI:** 10.1038/s42256-023-00652-2

**Published:** 2023-07-17

**Authors:** Alexandros Karargyris, Renato Umeton, Micah J. Sheller, Alejandro Aristizabal, Johnu George, Anna Wuest, Sarthak Pati, Hasan Kassem, Maximilian Zenk, Ujjwal Baid, Prakash Narayana Moorthy, Alexander Chowdhury, Junyi Guo, Sahil Nalawade, Jacob Rosenthal, David Kanter, Maria Xenochristou, Daniel J. Beutel, Verena Chung, Timothy Bergquist, James Eddy, Abubakar Abid, Lewis Tunstall, Omar Sanseviero, Dimitrios Dimitriadis, Yiming Qian, Xinxing Xu, Yong Liu, Rick Siow Mong Goh, Srini Bala, Victor Bittorf, Sreekar Reddy Puchala, Biagio Ricciuti, Soujanya Samineni, Eshna Sengupta, Akshay Chaudhari, Cody Coleman, Bala Desinghu, Gregory Diamos, Debo Dutta, Diane Feddema, Grigori Fursin, Xinyuan Huang, Satyananda Kashyap, Nicholas Lane, Indranil Mallick, FeTS Consortium, BraTS-2020 Consortium, AI4SafeChole Consortium, Pietro Mascagni, Virendra Mehta, Cassiano Ferro Moraes, Vivek Natarajan, Nikola Nikolov, Nicolas Padoy, Gennady Pekhimenko, Vijay Janapa Reddi, G. Anthony Reina, Pablo Ribalta, Abhishek Singh, Jayaraman J. Thiagarajan, Jacob Albrecht, Thomas Wolf, Geralyn Miller, Huazhu Fu, Prashant Shah, Daguang Xu, Poonam Yadav, David Talby, Mark M. Awad, Jeremy P. Howard, Michael Rosenthal, Luigi Marchionni, Massimo Loda, Jason M. Johnson, Spyridon Bakas, Peter Mattson

**Affiliations:** 1 IHU Strasbourg, Strasbourg, France.; 2 University of Strasbourg, Strasbourg, France.; 3 Dana-Farber Cancer Institute, Boston, MA, USA.; 4 Weill Cornell Medicine, New York, NY, USA.; 5 Harvard T.H. Chan School of Public Health, Boston, MA, USA.; 6 Massachusetts Institute of Technology, Cambridge, MA, USA.; 7 Intel, Santa Clara, CA, USA.; 8 Factored, Palo Alto, CA, USA.; 9 Nutanix, San Jose, CA, USA.; 10 Perelman School of Medicine, Philadelphia, PA, USA.; 11 University of Pennsylvania, Philadelphia, PA, USA.; 12 German Cancer Research Center, Heidelberg, Germany.; 13 University of Heidelberg, Heidelberg, Germany.; 14 MLCommons, San Francisco, CA, USA.; 15 Stanford University, Stanford, CA, USA.; 16 University of Cambridge, Cambridge, UK.; 17 Flower Labs, Hamburg, Germany.; 18 Sage Bionetworks, Seattle, WA, USA.; 19 Hugging Face, New York, NY, USA.; 20 Microsoft, Redmond, WA, USA.; 21 A*STAR, Singapore, Singapore.; 22 Supermicro, San Jose, CA, USA.; 23 Meta, Menlo Park, CA, USA.; 24 Stanford University School of Medicine, Stanford, CA, USA.; 25 Rutgers University, New Brunswick, NJ, USA.; 26 Landing.AI, Palo Alto, CA, USA.; 27 Red Hat, Raleigh, NC, USA.; 28 cKnowledge, Paris, France.; 29 OctoML, Seattle, WA, USA.; 30 Cisco, San Jose, CA, USA.; 31 IBM Research, San Jose, CA, USA.; 32 Tata Medical Center, Kolkata, India.; 33 Fondazione Policlinico Universitario A. Gemelli IRCCS, Rome, Italy.; 34 University of Trento, Trento, Italy.; 35 Write Choice, Florianópolis, Brazil.; 36 Google, Mountain View, CA, USA.; 37 University of Toronto, Toronto, Ontario, Canada.; 38 Vector Institute, Toronto, Ontario, Canada.; 39 Harvard University, Cambridge, MA, USA.; 40 NVIDIA, Santa Clara, CA, USA.; 41 Lawrence Livermore National Laboratory, Livermore, CA, USA.; 42 University of York, York, UK.; 43 John Snow Labs, Lewes, DE, USA.; 44 Harvard Medical School, Boston, MA, USA.; 45 fast.ai, San Francisco, CA, USA.; 46 University of Queensland, Brisbane, Queensland, Australia.; 47 Brigham and Women’s Hospital, Boston, MA, USA.; 48 Broad Institute of MIT and Harvard, Cambridge, MA, USA.; 49 These authors contributed equally: Alexandros Karargyris, Renato Umeton, Micah J. Sheller.; 50 These authors jointly supervised this work: Spyridon Bakas, Peter Mattson.

## Abstract

Medical artificial intelligence (AI) has tremendous potential to advance healthcare by supporting and contributing to the evidence-based practice of medicine, personalizing patient treatment, reducing costs, and improving both healthcare provider and patient experience. Unlocking this potential requires systematic, quantitative evaluation of the performance of medical AI models on large-scale, heterogeneous data capturing diverse patient populations. Here, to meet this need, we introduce MedPerf, an open platform for benchmarking AI models in the medical domain. MedPerf focuses on enabling federated evaluation of AI models, by securely distributing them to different facilities, such as healthcare organizations. This process of bringing the model to the data empowers each facility to assess and verify the performance of AI models in an efficient and human-supervised process, while prioritizing privacy. We describe the current challenges healthcare and AI communities face, the need for an open platform, the design philosophy of MedPerf, its current implementation status and real-world deployment, our roadmap and, importantly, the use of MedPerf with multiple international institutions within cloud-based technology and on-premises scenarios. Finally, we welcome new contributions by researchers and organizations to further strengthen MedPerf as an open benchmarking platform.

As medical artificial intelligence (AI) has begun to transition from research to clinical care^1–3^, national agencies around the world have started drafting regulatory frameworks to support and account for a new class of interventions based on AI models. Such agencies include the US Food and Drug Administration^4^, the European Medicines Agency^5^ and the Central Drugs Standard Control Organisation in India^6^. A key point of agreement for all regulatory agency efforts is the need for large-scale validation of medical AI models^7–9^ to quantitatively evaluate their generalizability.

Improving evaluation of AI models requires expansion and diversification of clinical data sourced from multiple organizations and diverse population demographics^1^. Medical research has demonstrated that using large and diverse datasets during model training results in more accurate models that are more generalizable to other clinical settings^10^. Furthermore, studies have shown that models trained with data from limited and specific clinical settings are often biased with respect to specific patient populations^11–13^; such data biases can lead to models that seem promising during development but have lower performance in wider deployment^14,15^.

Despite the clear need for access to larger and more diverse datasets, data owners are constrained by substantial regulatory, legal and public perception risks, high up-front costs, and uncertain financial return on investment. Sharing patient data presents three major classes of risk: (1) liability risk, due to theft or misuse; (2) regulatory constraints such as the Health Insurance Portability and Accountability Act or General Data Protection Regulation^16,17^; and (3) public perception risk, in using patient data that include protected health information that could be linked to individuals, compromising their privacy^18–25^. Sharing data also requires up-front investment to turn raw data into AI-ready formats, which comes with substantial engineering and organizational cost. This transformation often involves multiple steps including data collection, transformation into a common representation, de-identification, review and approval, licensing, and provision. Navigating these steps is costly and complex. Even if a data owner (such as a hospital) is willing to pay these costs and accept these risks, benefits can be uncertain for financial, technical or perception reasons. Financial success of an AI solution is difficult to predict and—even if successful—the data owner may see a much smaller share of the eventual benefit than the AI developer, even though the data owner may incur a greater share of the risk.

## Evaluation on global federated datasets

Here we introduce MedPerf^26^, a platform focused on overcoming these obstacles to broader data access for AI model evaluation. MedPerf is an open benchmarking platform that combines: (1) a lower-risk approach to testing models on diverse data, without directly sharing the data; with (2) the appropriate infrastructure, technical support and organizational coordination that facilitate developing and managing benchmarks for models from multiple sources, and increase the likelihood of eventual clinical benefit. This approach aims to catalyse wider adoption of medical AI, leading to more efficacious, reproducible and cost-effective clinical practice, with ultimately improved patient outcomes.

**Fig. 1 |:**
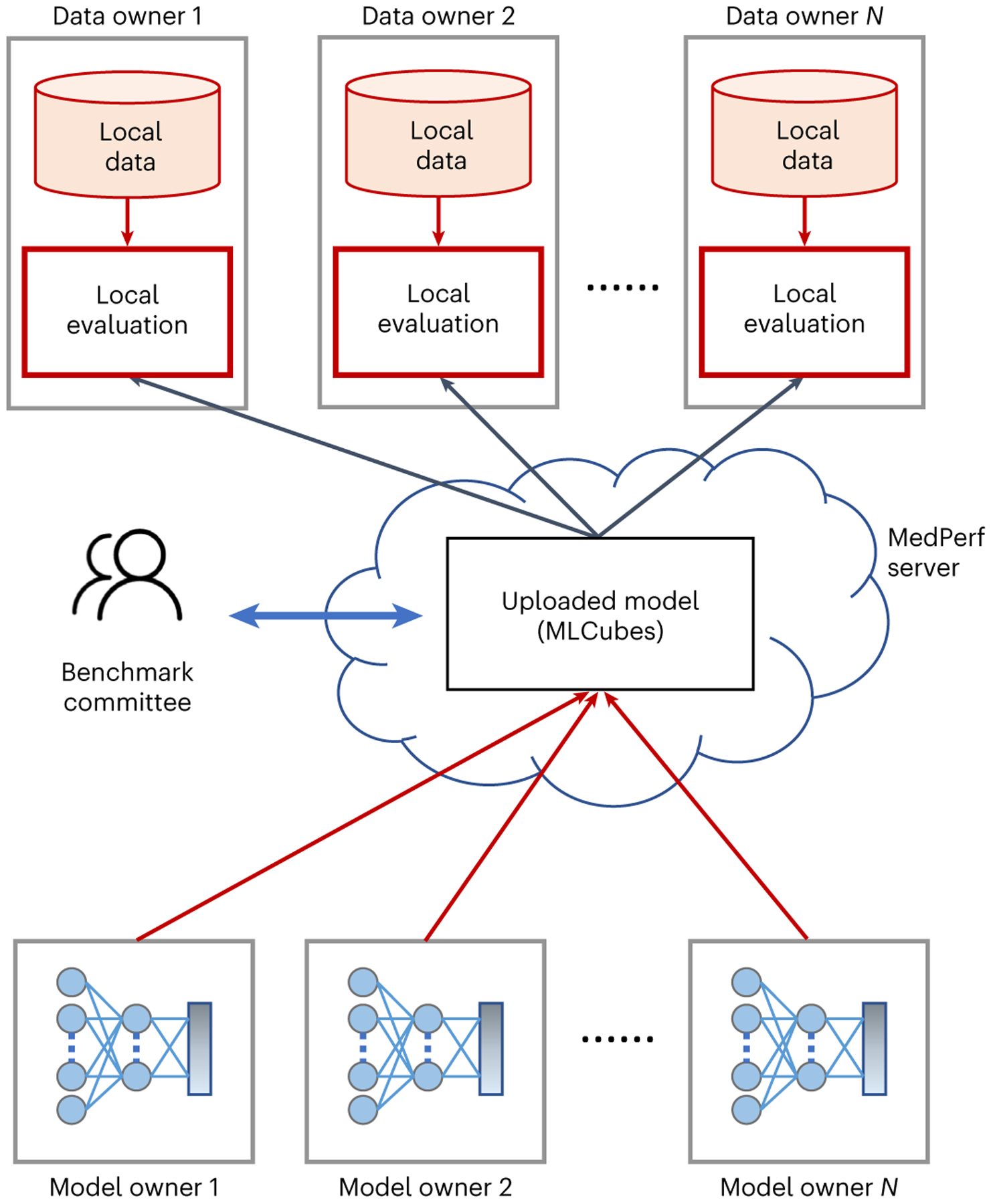
Federated evaluation on MedPerf. Machine learning models are distributed to data owners for local evaluation on their premises without the need or requirement to extract their data to a central location.

Our technical approach uses federated evaluation (Fig. 1), which aims to provide easy and reliable sharing of models among multiple data owners, for the purposes of evaluating these models against data owners’ data in locally controlled settings and enabling aggregate analysis of quantitative evaluation metrics. Importantly, by sharing trained AI models (instead of data) with data owners, and by aggregating only evaluation metrics, federated evaluation poses a much lower risk to patient data compared with federated training of AI models. Evaluation metrics generally yield orders of magnitude less information than model weight updates used in training, and the evaluation workflow does not require an active network connection during the workload, making it easier to determine the exact experiment outputs. Despite its promising features, federated evaluation requires submitting AI models to evaluation sites, which may pose a different type of risk^27,28^. Overall, our technology choices are aligned with the adoption growth federated approaches are experiencing in medicine and healthcare^2^.

MedPerf was created by a broad consortium of experts. The current list of direct contributors includes representatives from over 20 companies, 20 academic institutions and nine hospitals across thirteen countries and five continents. MedPerf was built upon the work experience that this group of expert contributors accrued in leading and disseminating past efforts such as (1) the development of standardized benchmarking platforms (such as MLPerf, for benchmarking machine learning training^29^ and inference across industries in a pre-competitive space^30^); (2) the implementation of federated learning software libraries such as the Open Federated Learning library^31^, NVIDIA FLARE, Flower by Flower Labs/University of Cambridge, and Microsoft Research FLUTE^32^; (3) the ideation, coordination and successful execution of computational competitions (also known as challenges) across dozens of clinical sites and research institutes (for example, BraTS^33^ and Federated Tumor Segmentation (FeTS)^34^; and (4) other prominent medical AI and machine learning efforts spanning multiple countries and healthcare specialties (such as oncology^3,29,35,36^ and COVID-19^37^).

MedPerf aims to bring the following benefits to the community: (1) consistent and rigorous methodologies to quantitatively evaluate performance of AI models for real-world use; (2) a technical approach that enables quantification of model generalizability across institutions, while aiming for data privacy and protection of model intellectual property; and (3) a community of experts to collaboratively design, operate and maintain medical AI benchmarks. MedPerf will also illuminate use cases in which better models are needed, increase adoption of existing generalizable models, and incentivize further model development, data annotation, curation and data access while preserving patient privacy.

## Results

**Fig. 2 |:**
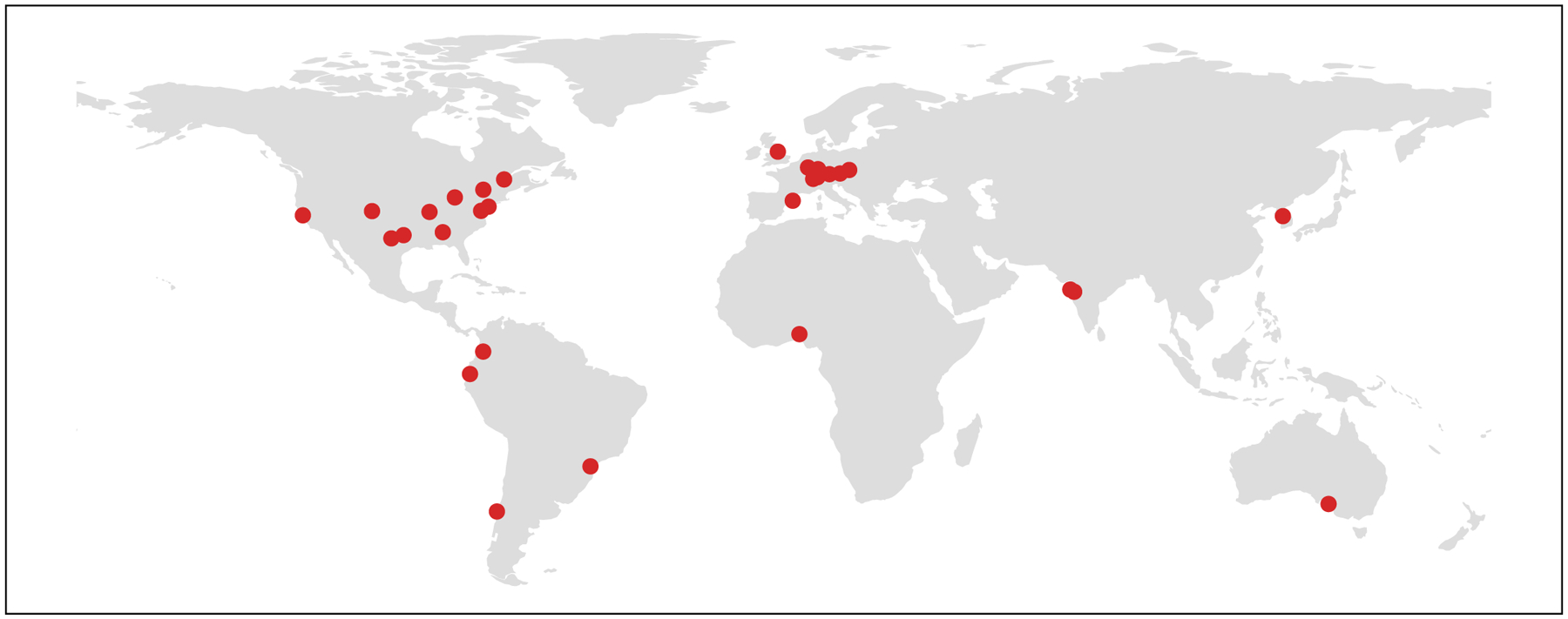
Geographical distribution of the FeTS collaborating sites in 2022. For the MICCAI FeTS 2022 challenge, our MedPerf platform facilitated the distribution, execution and collection of model results from 32 hospitals across Africa, North America, South America, Asia, Australia and Europe.

**Fig. 3 |:**
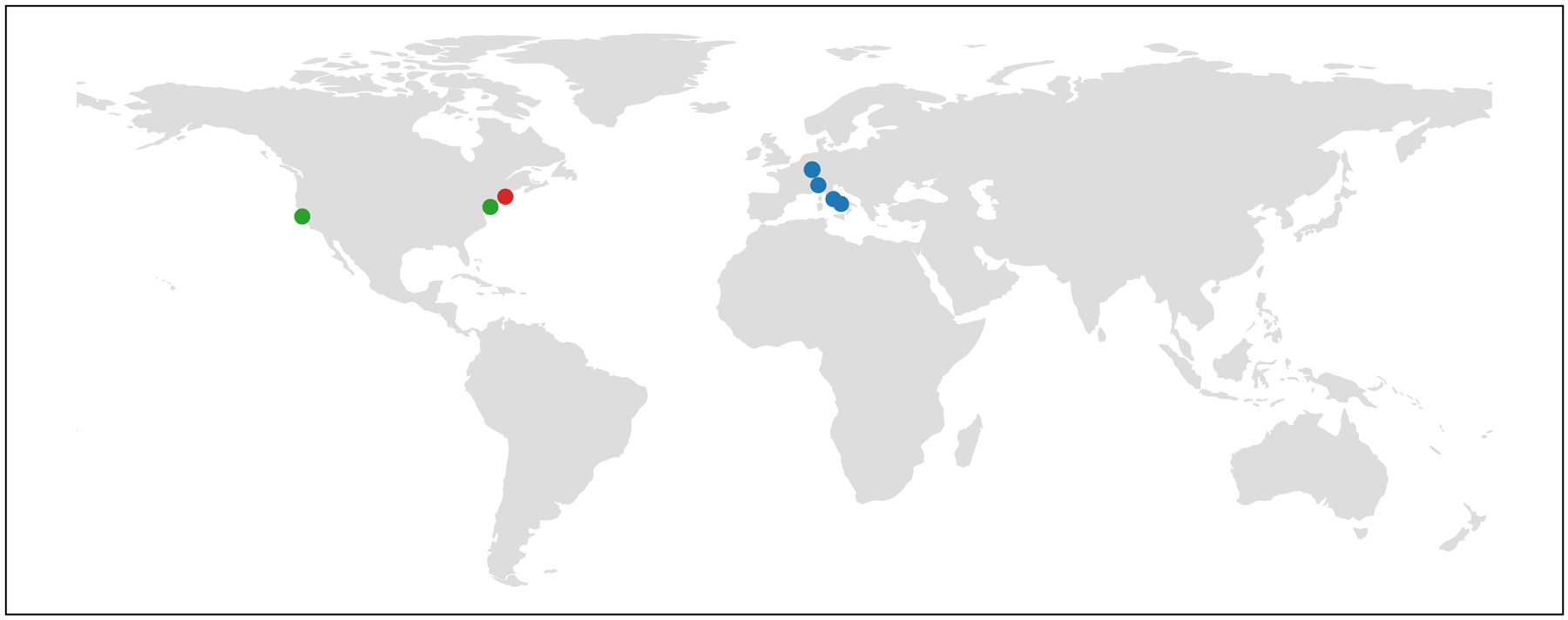
Locations of the data sources used in the pilot studies. The locations of the data sources used in the brain tumour segmentation (green), pancreas segmentation (red) and surgical workflow phase recognition (blue) pilot studies are shown.

MedPerf has already been used in a variety of settings including a chief use-case for the FeTS challenge^3,34,38^, as well as four academic pilot studies. In the FeTS challenge—the first federated learning challenge ever conducted—MedPerf successfully demonstrated its scalability and user-friendliness when benchmarking 41 models in 32 sites across six continents (Fig. 2). Furthermore, MedPerf was validated through a series of pilot studies with academic groups involved in multi-institutional collaborations for the purposes of research and development of medical AI models (Fig. 3). These studies included tasks on brain tumour segmentation (pilot study 1), pancreas segmentation (pilot study 2) and surgical workflow phase recognition (pilot 3), all of which are fully detailed in Supplementary Information. Collectively, all studies were intentionally designed to include a diverse set of clinical areas and data modalities to test MedPerf’s infrastructure adaptability. Moreover, the experiments included public and private datasets (pilot study 3), highlighting the technical capabilities of MedPerf to operate on private data. Finally, we performed benchmark experiments of MedPerf in the cloud to further test the versatility of the platform and pave the way to the benchmarking of private models; that is, models that are accessible only via an application programming interface (API), such as generative pre-trained transformers. All of the pilot studies used the default MedPerf server, whereas FeTS used its own MedPerf server. Each data owner (see Methods for a detailed role description) was registered with the MedPerf server. For the public datasets (pilot studies 1 and 2), and for the purposes of benchmarking, each data owner represented a single public dataset source. Each data owner prepared data according to the benchmark reference implementation and then registered the prepared data to the MedPerf server (see Methods). Finally, model MLCube containers (see Methods) comprising pretrained models were registered with the MedPerf server and evaluated on the data owners’ data. A detailed description for each benchmark—inclusive of data and source code—is provided in Supplementary Information.

We also collected feedback from FeTS and the pilots’ participating teams regarding their experience with MedPerf. The feedback was largely positive and highlighted the versatility of MedPerf, but also underlined current limitations, issues and enhancement requests that we are actively addressing. Mainly, technical documentation on MedPerf was reported to be limited, creating an extra burden to users. Since then, the documentation has been extensively revamped^39^. Second, the dataset information provided to users was limited, requiring benchmark administrators to manually inspect model–dataset associations before approval. Finally, benchmark error logging was minimal, thus increasing debugging effort. The reader is advised to visit the MedPerf issue tracker for a more complete and up-to-date list of open and closed issues, bugs and feature requests^40^.

### MedPerf roadmap

Ultimately, MedPerf aims to deliver an open-source software platform that enables groups of researchers and developers to use federated evaluation to provide evidence of generalized model performance to regulators, healthcare providers and patients. We started with specific use cases with key partners (that is, the FeTS challenge and pilot studies), and we are currently working on general purpose evaluation of healthcare AI through larger collaborations, while extending best practices into federated learning. In Table 1, we review the necessary next steps, the scope of each step, and the current progress towards developing this open benchmarking ecosystem. Beyond the ongoing improvement efforts described here, the philosophy of MedPerf involves open collaborations and partnerships with other well-established organizations, frameworks and companies.

One example is our partnership with Sage Bionetworks; specifically, several ad-hoc components required for MedPerf-FeTS integration were built upon the Synapse platform^41^. Synapse supports research data sharing and can be used to support the execution of community challenges. These ad-hoc components included: (1) creating a landing page for the benchmarking competition^38^, which contained all instructions as well as links to further material; (2) storing the open models in a shared place; (3) storing the demo data in a similarly accessible place; (4) private and public leaderboards; and (5) managing participant registration and competition terms of use. A notable application of Synapse has been supporting DREAM challenges for biomedical research since 2007^42^. The flexibility of Synapse allows for privacy preserving model-to-data competitions^43,44^ that prevent public access to sensitive data. With MedPerf, this concept can take on another dimension by ensuring the independent security of data sources. As medical research increasingly involves collecting more data from larger consortia, there will be greater demands on computing infrastructure. Research fields in which community data competitions are popular stand to benefit from federated learning frameworks that are capable of learning from data collected worldwide.

To increase the scalability of MedPerf, we also partnered with Hugging Face to leverage its hub platform^45^, and demonstrated how new benchmarks can use the Hugging Face infrastructure. In the context of Hugging Face, MedPerf benchmarks can have associated organization pages on the Hugging Face Hub, where benchmark participants can contribute models, datasets and interactive demos (collectively referred to as artifacts). The Hugging Face Hub can also facilitate automatic evaluation of models and provide a leaderboard of the best models based on benchmark specifications (for example, the PubMed summarization task^46^). Benefits of using the Hugging Face Hub include the fact that artifacts can be accessed from Hugging Face’s popular open-source libraries, such as datasets^47^, transformers^48^ and evaluation^49^. Furthermore, artifacts can be versioned, documented with detailed datasets/model cards, and designated with unique digital object identifiers. The integration of MedPerf and Hugging Face demonstrates the extensibility of MedPerf to popular machine learning development platforms.

To enable wider adoption, MedPerf supports popular machine learning libraries that offer ease of use, flexibility and performance. Popular graphical user interfaces and low-code frameworks such as MONAI^50^, Lobe^51^, KNIME^52^ and fast.ai^53^ have substantially lowered the difficulty of developing machine learning pipelines. For example, the open-source fast.ai library has been popular in the medical community due to its simplicity and flexibility to create and train medical computer vision models in only a few lines of code.

Finally, MedPerf can also support private AI models or AI models available only through API, such as OpenAI GPT-4 (ref. 54), Hugging Face Inference Endpoints^55^ and Epic Cognitive Computing (https://galaxy.epic.com/?#Browse/page=1!68!715!100031038). As these private-model APIs effectively run on protected health information data, we see a lower barrier to entry in their adoption via Azure OpenAI Services, Epic Cognitive Computing and similar services that guarantee compliance of the API (for example, Health Insurance Portability and Accountability Act or General Data Protection Regulation). Although this adds a layer of complexity, it is important that MedPerf is compatible with these API-only AI solutions.

Although the initial uses of MedPerf were in radiology and surgery, MedPerf can easily be used in other biomedical tasks such as computational pathology, genomics, natural language processing (NLP), or the use of structured data from the patient medical record. Our catalogue of examples is regularly updated^56^ to highlight various use cases. As data engineering and availability of validated pretrained models are common pain points, we plan to develop more MedPerf examples for the specialized, low-code libraries in computational pathology, such as PathML^57^ or SlideFlow^58^, as well as Spark NLP^59^, to fill the data engineering gap and enable access to state-of-the-art pretrained computer vision and NLP models. Furthermore, our partnership with John Snow Labs facilitates integration with the open-source Spark NLP and the commercial Spark NLP for Healthcare^60–62^ within MedPerf.

The MedPerf roadmap described here highlights the potential of future platform integrations to bring additional value to our users and establish a robust community of researchers and data providers.

### Related work

The MedPerf effort is inspired by past work, some of which is already integrated with MedPerf, and other efforts we plan to integrate as part of our roadmap. Our approach to building on the foundation of related work has four distinct components. First, we adopt a federated approach to data analyses, with the initial focus on quantitative algorithmic evaluation toward lowering barriers to adoption. Second, we adopt standardized measurement approaches to medical AI from organizations—including the Special Interest Group on Biomedical Image Analysis Challenges of MICCAI^63^, the Radiological Society of North America, the Society for Imaging Informatics in Medicine, Kaggle, and Synapse—and we generalize these efforts to a standard platform that can be applied to many problems rather than focus on a specific one^14,64–67^. Third, we leverage the open, community-driven approach to benchmark development successfully employed to accelerate hardware development, through efforts such as MLPerf/MLCommons and SPEC^68^, and apply it to the medical domain. Finally, we push towards creating shared best practices for AI, as inspired by efforts such as MLflow^69^, Kubeflow for AI operations^70^, MONAI^50^, Substra^71^, Fed-BioMed^72^, the Joint Imaging Platform from the German Cancer Research Center^73^, and the Generally Nuanced Deep Learning Framework^74,75^ for medical models. And we acknowledge and take inspiration from existing efforts such as the Breaking Barriers to Health Data project led by the World Economic Forum^10^.

## Discussion

MedPerf is a benchmarking platform designed to quantitatively evaluate AI models ‘in the wild,’ considering unseen data from out-of-sample distinct sources, and thereby helping address inequities, bias and fairness in AI models. Our initial goal is to provide medical AI researchers with reproducible benchmarks based on diverse patient populations to assist healthcare algorithm development. Robust well-defined benchmarks have shown their impact in multiple industries^76,77^ and such benchmarks in medical AI have similar potential to increase development interest and solution quality, leading to patient benefit and growing adoption while addressing underserved and underrepresented patient populations. Furthermore, with our platform we aim to advance research related to data utility, model utility, robustness to noisy annotations and understanding of model failures. Wider adoption of such benchmarking standards will substantially benefit their patient populations. Ultimately, standardizing best practices and performance evaluation methods will lead to highly accurate models that are acceptable to regulatory agencies and clinical experts, and create momentum within patient advocacy groups whose participation tends to be underrepresented^78^. By bringing together these diverse groups—starting with AI researchers and healthcare organizations, and by building trust with clinicians, regulatory authorities and patient advocacy groups—we envision accelerating the adoption of AI in healthcare and increasing clinical benefits to patients and providers worldwide. Notably, our MedPerf efforts are in complete alignment with the Blueprint for an AI Bill of Rights recently published by the US White House^79^ and would serve well the implementation of such a pioneering bill.

However, we cannot achieve these benefits without the help of the technical and medical community. We call for the following:

- Healthcare stakeholders to form benchmark committees that define specifications and oversee analyses.
- Participation of patient advocacy groups in the definition and dissemination of benchmarks.
- AI researchers to test this end-to-end platform and use it to create and validate their own models across multiple institutions around the globe.
- Data owners (for example, healthcare organizations, clinicians) to register their data in the platform (no data sharing required).
- Data model standardization efforts to enable collaboration between institutions, such as the OMOP Common Data Model^80,81^, possibly leveraging the highly multimodal nature of biomedical data^82^.
- Regulatory bodies to develop medical AI solution approval requirements that include technically robust and standardized guidelines.

We believe open, inclusive efforts such as MedPerf can drive innovation and bridge the gap between AI research and real-world clinical impact. To achieve these benefits, there is a critical need for broad collaboration, reproducible, standardized and open computation, and a passionate community that spans academia, industry, and clinical practice. With MedPerf, we aspire to bring such a community of stakeholders together as a critical step toward realizing the grand potential of medical AI, and we invite participation at ref. 26.

## Methods

In this section we describe the structure and functionality of MedPerf as an open benchmarking platform for medical AI. We define a MedPerf benchmark, describe the MedPerf platform and MLCube interface at a high level, discuss the user roles required to successfully operate such a benchmark, and provide an overview of the operating workflow. The reader is advised to refer to ref. 39 for up-to-date, extensive documentation.

The technical objective of the MedPerf platform is threefold: (1) facilitate delivery and local execution of the right code to the right private data owners; (2) facilitate coordination and organization of a federation (for example, discovery of participants, tracking of which steps have been run); and (3) store experiment records, such as which steps were run by whom, and what the results were, and to provide the necessary traceability to validate the experiments.

The MedPerf platform comprises three primary types of components:

1. The MedPerf server, which is used to define, register and coordinate benchmarks and users, as well as record benchmark results. It uses a database to store the minimal information necessary to coordinate federated experiments and support user management, such as: how to obtain, verify and run MLCubes; which private datasets are available to—and compatible with—a given benchmark (commonly referred to as association); and which models have been evaluated against which datasets, and under which metrics. No code assets or datasets are stored on the server (see the database SQL files at ref. 83).
2. The MedPerf client, which is used to interact with the MedPerf Server for dataset/MLCube checking and registration, and to perform benchmark experiments by downloading, verifying and executing MLCubes.
3. The benchmark MLCubes (for example, the AI model code, performance evaluation code, data quality assurance code), which are hosted in indexed container registries (such as DockerHub, Singularity Cloud and GitHub).

In a federated evaluation platform, data are always accessed and analysed locally. Furthermore, all quantitative performance evaluation metrics (that is, benchmark results) are uploaded to the MedPerf Server only if approved by the evaluating site. The MedPerf Client provides a simple interface—common across all benchmark code/models—for the user to download and run any benchmark.

### MedPerf benchmarks

For the purposes of our platform, a benchmark is defined as a bundle of assets that enables quantitative evaluation of the performance of AI models for a specific clinical task, and consists of the following major components:

1. Specifications: precise definition of the (1) clinical setting (for example, the task, medical use-case and potential impact, type of data and specific patient inclusion criteria) on which trained AI models are to be evaluated; (2) labelling (annotation) methodology; and (3) performance evaluation metrics.
2. Dataset preparation: code that prepares datasets for use in the evaluation step and can also assess prepared datasets for quality control and compatibility.
3. Registered datasets: a list of datasets prepared by their owners according to the benchmark criteria and approved for evaluation use by their owners.
4. Registered models: a list of AI models to execute and evaluate in this benchmark.
5. Evaluation metrics: an implementation of the quantitative performance evaluation metrics to be applied to each registered model’s outputs.
6. Reference implementation: an example of a benchmark submission consisting of an example model code, the performance evaluation metric component described above, and publicly available de-identified or synthetic sample data.
7. Documentation: documentation for understanding and using the benchmark and its aforementioned components.

### MedPerf and MLCubes

MLCube is a set of common conventions for creating secure machine learning/AI software container images (such as Docker and Singularity) compatible with many different systems. MedPerf and MLCube provide simple interfaces and metadata to enable the MedPerf client to download and execute a MedPerf benchmark.

In MedPerf MLCubes contain code for the following benchmark assets: dataset preparation, registered models, performance evaluation metrics and reference implementation. Accordingly, we define three types of MedPerf MLCubes: the data preparation MLCube, model MLCube, and evaluation metrics MLCube.

The data preparation MLCube prepares the data for executing the benchmark, checks the quality and compatibility of the data with the benchmark (that is, association), and computes statistics and metadata for registration purposes. Specifically, it’s interface exposes three functions:

- Prepare: transforms input data into a consistent data format compatible with the benchmark models.
- Sanity check: ensures data integrity of the prepared data, checking for anomalies and data corruption.
- Statistics: computes statistics on the prepared data; these statistics are displayed to the user and, given user consent, uploaded to the MedPerf server for dataset registration.

The model MLCube contains a pretrained AI model to be evaluated as part of the benchmark. It provides a single function, infer, which computes predictions on the prepared data output by the data preparation MLCube. In the future case of API-only models, this would be the container hosting the API wrapper to reach the private model.

The evaluation metrics MLCube computes metrics on the model predictions by comparing them against the provided labels. It exposes a single ‘evaluate’ function, which receives as input the locations of the predictions and prepared labels, computes the required metrics, and writes them to a results file. Note that the results file is uploaded to the server by the MedPerf only after being approved by the owner.

With MLCubes, the infrastructure software can efficiently interact with models, which means it can be implemented in various frameworks, run on different hardware platforms, and leverage common software tools for validating proper secure implementation practices (for example, CIS Docker Benchmarks).

### Benchmarking user roles

We have identified four primary roles in operating an open benchmark platform, as outlined in Table 2. Depending on the rules of a benchmark, in many cases, a single organization may participate in multiple roles, and multiple organizations may share any given role. Beyond these roles, the long term success of medical AI benchmarking requires strong participation of organizations that create and adopt appropriate community standards for interoperability; for example, Vendor Neutral Archives^84,85^, DICOM^80^, NIFTI^86^, OMOP^80,81^, PRISSMM^87^ and HL7/FHIR^88^.

### Benchmarking workflow

**Fig. 4 |:**
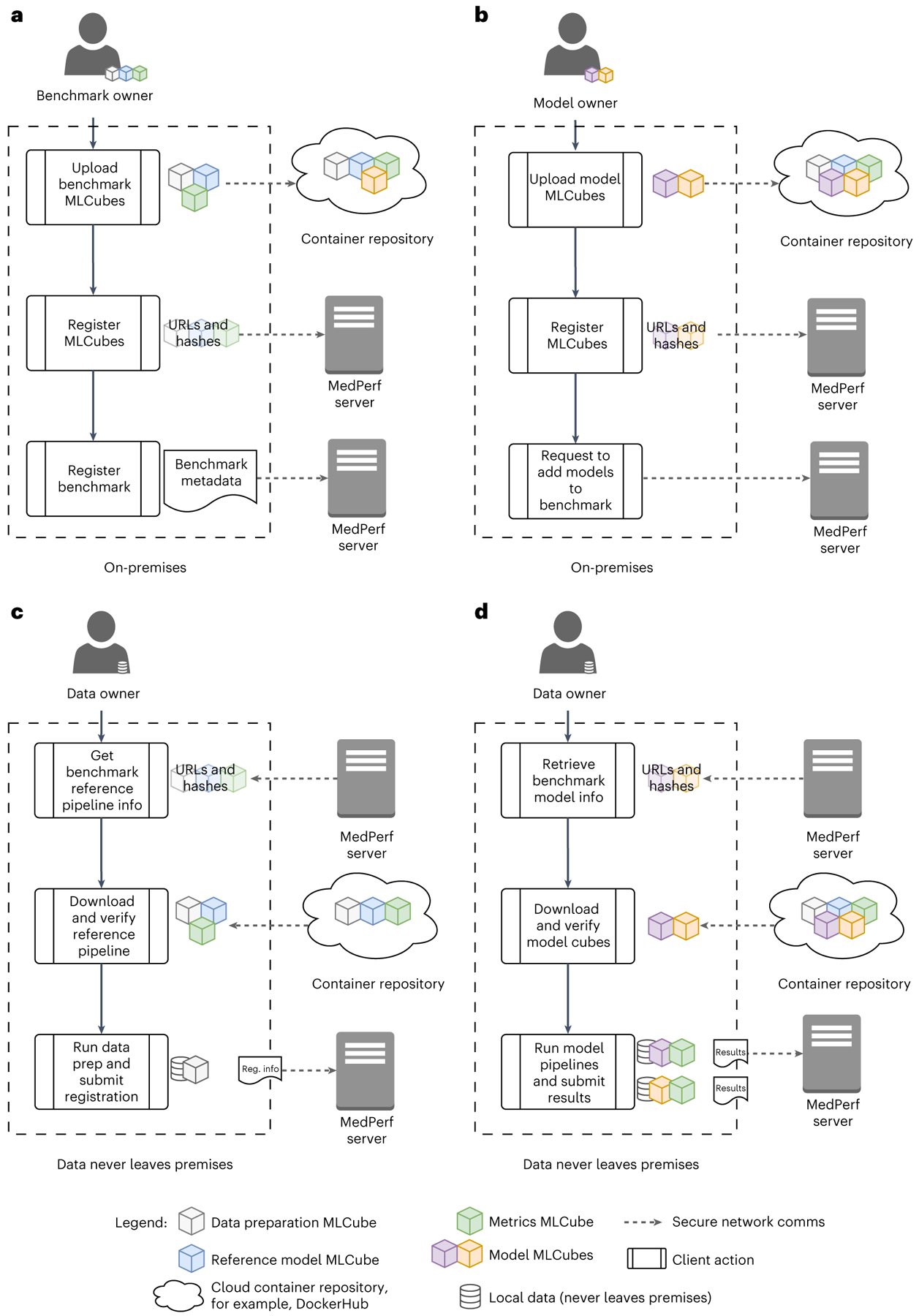
Description of MedPerf workflows. All user actions are performed via the MedPerf client, except uploading to container repositories. **a**, Benchmark registration by the benchmark committee: the committee uploads the data preparation, reference model and evaluation metrics MLCubes to a container repository and then registers them with the MedPerf Server. The committee then submits the benchmark registration, including required benchmark metadata. **b**, Model registration by the model owner: the model owner uploads the model MLCube to a container repository and then registers it with the MedPerf Server. They may then request inclusion of models in compatible benchmarks. **c**, Dataset registration by the data owner: the data owner downloads the metadata for the data preparation, reference model and evaluation metrics MLCubes from the MedPerf server. The MedPerf client uses these metadata to download and verify the corresponding MLCubes. The data owner runs the data preparation steps and submits the registration output via the data preparation MLCube to the MedPerf server. **d**, Execution of benchmark: the data owner downloads the metadata for the MLCubes used in the benchmark. The MedPerf client uses these metadata to download and verify the corresponding MLCubes. For each model, the data owner executes the model-to-evaluation-metrics pipeline (that is, the model and evaluation metrics MLCubes) and uploads the results files output by the evaluation metrics MLCube to the MedPerf server. No patient data are uploaded to the MedPerf server.

Our open benchmarking platform, MedPerf, uses the workflow depicted in Fig. 4 and outlined in Table 3. All of the user actions in the workflow can be performed via the MedPerf client, with the exception of uploading MLCubes to cloud-hosted registries (for example, DockerHub, Singularity Cloud), which is performed independently.

#### Establishing a benchmark committee.

The benchmarking process starts with establishing a benchmark committee (for example, challenge organizers, clinical trial organizations, regulatory authorities and charitable foundation representatives), which identifies a problem for which an effective AI-based solution can have a clinical impact.

#### Recruiting data and model owners.

The benchmark committee recruits data owners researchers, AI vendors) either by inviting trusted parties or by making an open call for participation, such as a computational healthcare challenge. The recruitment process can be considered as an open call process for the data and model owners to register their contribution and benchmark intent. A higher number of recruited dataset providers may result in larger diversity on a global scale.

#### MLCubes and benchmark submission.

To register the benchmark on the MedPerf platform, the benchmark committee first needs to submit the three reference MLCubes: data preration MLCube, model MLCube and evaluation metrics MLCube. After submitting these three MLCubes, the benchmark committee may initiate a benchmark. Once the benchmark is submitted, the MedPerf administrator must approve it before it becomes available to platform users. This submission process is presented in Fig. 4a.

#### Submitting and associating additional models.

With the benchmark approved by the MedPerf administrator, model owners can submit their own model MLCubes and request an association with the benchmark. This association request executes the benchmark locally with the given model to ensure compatibility. If the model successfully passes the compatibility test, and its association is approved by the benchmark committee, then it becomes part of the benchmark. The association process of model owners is shown in Fig. 4b.

#### Dataset preparation and association.

Data owners that would like to participate in the benchmark can prepare their own datasets, register them and associate them with the benchmark. Data owners can run the data preparation MLCube so that they can extract, preprocess, label and review their dataset in accordance with their legal and ethical compliance requirements. If data preparation is successful, the dataset has successfully passed the compatibility test. Once association is approved by the benchmark committee, then the dataset is registered with MedPerf and associated with that specific benchmark. Figure 4c shows the dataset preparation and association process for data owners.

#### Executing the benchmark.

Once the benchmark, datasets and models are registered to the benchmarking platform, the benchmark committee notifies data owners that models are available for benchmarking, thus they can generate results by running a model on their local data. This execution process is shown in Fig. 4d. The procedure retrieves the specified Model MLCube and runs it with the indicated prepared dataset to generate predictions. The model MLCube executes the machine learning inference task to generate predictions based on the prepared data. Finally, the evaluation metrics MLCube is retrieved to compute metrics on the predictions. Once results are generated, the data owner may approve and submit them to the platform and thus finalize the benchmark execution on their local data.

### Privacy considerations

The current implementation of MedPerf focuses on preserving privacy of the data used to evaluate models; however, privacy of the original training data is currently out of scope, and we leave privacy solutions to the model owners (for example, training with differential privacy and out-of-band encryption mechanisms).

However, privacy is of utmost importance to us. Hence future versions of MedPerf will include features that support model privacy and possibly a secure MedPerf container registry. We acknowledge that model privacy not only helps with intellectual property protection, but also mitigates model inversion attacks on data privacy, wherein a model is used to reconstruct its training data. Although techniques such as differential privacy, homomorphic encryption, file access controls and trusted execution environments can all be pursued and applied by the model and data owners directly, MedPerf will facilitate various techniques (for example, authenticating to private container repositories, storing hardware attestations, execution integrity for the MedPerf client itself) to strengthen privacy in models and data while lowering the burden to all involved.

From an information security and privacy perspective, no technical implementation should fully replace any legal requirements or obligations for the protection of data. MedPerf’s ultimate objectives are to: (1) streamline the requirements process for all parties involved in medical AI benchmarking (patients, hospitals, benchmark owners, model owners and so on) by adopting standardized privacy and security technical provisions; and (2) disseminate these legal provisions in a templated terms and conditions document (that is, the MedPerf Terms and Use Agreement), which leverages MedPerf technical implementation to achieve a faster and more repeatable process. As of today, hospitals that want to share data typically require a data transfer agreement or data use agreement. Achieving such agreements can be time-consuming, often taking several months or more to complete. With MedPerf most technical safeguards will be agreed on by design and thus immutable, allowing the templated agreement terms and conditions to outline the more basic and common-sense regulatory provisions (for example, prohibiting model reverse engineering or exfiltrating data from pretrained models), and enabling faster legal handshakes among involved parties.

## Supplementary Material

Supplementary material

## Figures and Tables

**Table 1 |:** MedPerf roadmap stages, scopes, and corresponding details for each stage

Roadmap stage	Scope: STATUS	Details
Design	Design for an open medical benchmarking platform—completed.	MedPerf was designed by the non-profit MLCommons Association. MLCommons brings together engineers and academics globally to make AI better for all; they have already created and host the MLPerf benchmark suites for AI performance (as measured by speed-up, electrical consumption and so on).
Implementation of platform (alpha release)	Phase 1: single-system proof-of-concept—completed.	Implement and demonstrate technicaL approach using public data and open-source modeLs on a single system that simulates multiple systems (which eliminates platform incompatibility and communication issues).
	Phase 2: distributed proof-of-concept—compLeted.	Implement and demonstrate technical approach using public data and open-source models communicating across the internet on multiple systems belonging to potential data and model owners.
Improvements of platform (transition from alpha to beta release)	Model protection: in development federated learning capability—in development.	Identify and develop best practices for model intellectual protection. Build upon common federated learning frameworks. Integrate and propose best practices related to federated learning in medical AI.
Implementation and evaluation of sample benchmarks	Brain tumour segmentation-completed. Pancreas segmentation—completed Surgical phase recognition—completed.	We chose these motivating problems because they: (1) affect a large, global patient population and represent a substantial opportunity for clinical impact; (2) have high-potential AI solutions; and (3) have public datasets and open-source models in development.
Deployment	Phase 1: beta release—completed.	Selected number of benchmarking efforts using non-public data—chief use-case: FeTS challenge.
	Phase 2: wide-scale release—ongoing.	Open to all qualified benchmarking efforts.

**Table 2 |:** Benchmarking user roles and responsibilities

Role name	Role definition	Role responsibilities
Benchmark committee	Benchmark committee incLudes regulatory bodies, groups of experts (for example, clinicians, patient representative groups), and data or model owners wishing to drive evaluation of their model or data.	Authors the benchmark, manages all benchmark assets, and produces some assets (for example, dataset preparation). Recruits model owners and data owners, makes an open benchmark for model owners and approves applicants. Controls access to the aggregated statistical results.
Data owner	Data owners may include hospitals, medical practices, research organizations and healthcare insurance providers that ‘own’ medical data, register medical data and execute benchmark requests.	Registers data with benchmarking platform. Performs data labelling. Downloads and executes a data preparation processor to prepare data. Downloads and periodically uses platform client to approve and serve requests, and to approve and upload results to or from benchmarking platform.
Model owner	Model owners include AI researchers and software vendors that own a trained medical AI model and want to evaluate its performance.	Registers model with benchmarking platform Views results of their model on the benchmark Has the option to approve sharing of results of that benchmark with other model/data owners or the public if allowed by benchmark group
Platform provider	Organizations such as MLCommons, which operate a platform that enables benchmark groups to run benchmarks by connecting data owners with model owners.	Manages user accounts and provides a website for registering and discovering benchmarks, datasets, models, and for overall workflow management Coordinates active benchmarks by sending requests, aggregating results and managing result access

**Table 3 |:** Benchmarking workflow, steps and interconnections with roles

	Workflow step	Objective
1	Define and register benchmark	The benchmarking process starts with establishing a benchmark committee of healthcare stakeholders: healthcare organizations, clinical experts, AI researchers and patient advocacy groups. Benchmark committee identifies a clinical problem for which an effective AI-based solution can have a substantial clinical impact. Benchmark committee registers the benchmark on the platform and provides the benchmark assets (see ‘MedPerf Benchmarks’).
2	Recruit data owners	Benchmark committee recruits data and model owners either by inviting trusted parties or by making an open call for participation. Dataset owners are recruited to maximize aggregate dataset size and diversity on a global scale. Many benchmarking efforts may initially focus on data providers with existing agreements.
	Prepare and register datasets	In coordination with the benchmark committee, dataset owners are responsible for data preparation (that is, extraction, preprocessing, labelling, reviewing for legal/ethical compliance). Once the data are prepared and approved by the data owner, the dataset can be registered with the benchmarking platform.
3	Recruit model owners	Model owners modify the benchmark reference implementation. To enable consistent execution on data owner systems, the solutions are packaged inside of MLCube containers. Model owners must conduct appropriate legal and ethical review before submission of a solution for evaluation.
	Prepare and register models	Once implemented by the model owner and approved by the benchmark committee, the model can be registered on the platform.
4	Execute benchmarks	Once the benchmark, dataset and models are registered to the benchmarking platform, the platform notifies the data owners that models are available for benchmarking. The data owner runs a benchmarking cLient that downloads available models, reviews and approves models for safety, and then approves execution. Once execution is completed, the data owner reviews and approves upload of the results to the benchmark platform.
5	Release results	Benchmark results are aggregated by the benchmarking platform and shared per the policy specified by the benchmark committee, following data owners’ approval.

## Data Availability

All datasets used here are available in public repositories except for: (1) the Surgical Workflow Phase Recognition benchmark (pilot study 3), which used privately held surgical video data, and (2) the test dataset of the FeTS challenge, which was also private. Users can access each study’s dataset through the following links: FeTS challenge^38^; pilot study 1—brain tumour segmentation (https://www.med.upenn.edu/cbica/brats2020/data.html); pilot study 2—pancreas segmentation (https://www.synapse.org/#!Synapse:syn3193805 and https://wiki.cancerimagingarchive.net/display/Public/Pancreas-CT); and pilot study 4—cloud experiments (https://stanfordmlgroup.github.io/competitions/chexpert/).
