# Supplementary material for: Federated benchmarking of medical artificial intelligence with MedPerf
Source: Nat Mach Intell. Author manuscript; Available in PMC 2024 May 3. (PMC11068064; doi:10.1038/s42256-023-00652-2)
Supplement: Supplementary material [file NIHMS1940400-supplement-Supplementary_material.pdf]

nature machine intelligence

Article https://doi.org/10.1038/s42256-023-00652-2

Federated benchmarking of medical artificial intelligence with MedPerf

In the format provided by the authors and unedited

Supplementary Material

Table of Contents

1 Supplementary Method Material **2**
1.1 Details of MedPerf Benchmarks 2
1.1.1 Chief use-case: MICCAI FeTS Challenge 2
1.1.2 Pilot Study 1 - Brain Tumor Segmentation 3
1.1.2.1 Participating Institutions 3
1.1.2.2 Clinical Task 3
1.1.2.3 Data Description 3
1.1.3 Benchmark Assets 4
1.1.3.1 Benchmark Results 4
1.1.3.2 Limitations & Observations shared by Pilot Study Participants 5
1.1.4 Pilot Study 2 - Pancreas Segmentation 5
1.1.4.1 Participating Institutions 5
1.1.4.2 Clinical Task 6
1.1.4.3 Data Description 6
1.1.5 Benchmark Assets 6
1.1.5.1 Benchmark Results 7
1.1.5.2 Limitations & Observations shared by Pilot Study Participants 7
1.1.6 Pilot Study 3 - Surgical Workflow Phase Recognition 8
1.1.6.1 Participating institutions 8
1.1.6.2 Clinical Task 8
1.1.6.3 Data description 8
1.1.6.4 Benchmark Assets 9
1.1.6.5 Benchmark Results 9
1.1.6.6 Limitations & Observations shared by Pilot Study Participants 10
1.2 Pilot Study 4 - Cloud Experiments 10
2 Consortia **12**
2.1 AI4SafeChole Consortium 12
2.2 BraTS-2020 Consortium 12
2.3 CHAOS Consortium 12
2.4 FeTS Consortium 12
3 Supplementary References **13**

1 Supplementary Method Material

1.1 Details of MedPerf Benchmarks

1.1.1 Chief use-case: MICCAI FeTS Challenge

MedPerf was publicly used for the orchestration of the Federated Tumor Segmentation (FeTS, https://miccai2022.fets.ai) Challenge [1], the results of which were reported at the Annual Scientific Conference of the Medical Image Computing and Computer Assisted Interventions (MICCAI) 2022. The FeTS Challenge is the first federated learning challenge ever proposed across all domains. MedPerf was used to orchestrate the distribution, execution, and quantitative performance evaluation of AI models in out-of-sample data around the globe (FeTS challenge task 2, https://www.synapse.org/fets), i.e., sites that have not contributed any patient data in the dataset used by the model owners to train their AI models.

From a technical point of view, the FeTS Challenge Organizing Committee represented the *Benchmark Committee* (according to MedPerf's terminology), which 1) hosted an instance of the MedPerf Server on https://fets.medperf.org, 2) provided the benchmark's reference implementation (i.e., Data Preparation, Model, Evaluation Metrics), and 3) created a Benchmark on the MedPerf registry that served as its federated evaluation.

Overall, a total of 41 models were part of the FeTS federated evaluation, 5 of which were implemented by the challenge participants, and 36 were adapted by the FeTS Organizers from the BraTS 2021 Challenge [2]. All 41 models were submitted via MedPerf by the FeTS Challenge Organizers. MedPerf facilitated the distribution, execution, and collection of model results from 33 sites from Africa, North America, South America, Asia, Australia, and Europe (Figure 3 in main text). On each institution the MedPerf client was installed and it was used by local facilitators to execute on-site all models related to the benchmark. Out of 1,312 benchmark results, 770 were gathered directly through MedPerf while the remaining ones were not gathered during the challenge's lifecycle, either because of time constraints on the collaborator's side or issues that couldn't be covered in the expected amount of time (e.g GPU incompatibility issues, Singularity MLCubes execution, duplicate dataset identification numbers) . Local facilitators from multiple institutions (i.e., *Data Owners* according to MedPerf's

terminology) expressed their improved experience during the FeTS 2022 challenge when using MedPerf compared to the FeTS 2021 iteration, which did not use MedPerf but a custom evaluation platform. Specific improvements included substantial reduction of their time and effort in facilitating model retrieval, experiment execution, and results submission.

1.1.2 Pilot Study 1 - Brain Tumor Segmentation

1.1.2.1 Participating Institutions

- University of Pennsylvania, Philadelphia, USA
- Perelman School of Medicine at the University of Pennsylvania, Philadelphia, USA
- University of San Francisco, San Francisco, CA, USA

1.1.2.2 Clinical Task

Gliomas are highly heterogeneous across their molecular, phenotypical, and radiological landscape. Their radiological appearance is described by different sub-regions comprising 1) the “enhancing tumor” (ET), 2) the gross tumor, also known as the “tumor core” (TC), and 3) the complete tumor extent also referred to as the “whole tumor” (WT). ET is described by areas that show hyper-intensity in T1Gd when compared to T1, but also when compared to ”healthy” white matter in T1Gd. The TC describes the bulk of the tumor, which is what is typically resected. The TC entails the ET, as well as the necrotic (fluid-filled) parts of the tumor. The appearance of the necrotic (NCR) tumor core is typically hypo-intense in T1Gd when compared to T1. The WT describes the complete extent of the disease, as it entails the TC and the peritumoral edematous/infiltrated tissue (ED), which is typically depicted by abnormal hyper-intense signal in T2-FLAIR. These scans, with accompanying manually approved labels by expert neuroradiologists for these sub-regions, are provided in the International Brain Tumor Segmentation (BraTS) challenge data[2].

1.1.2.3 Data Description

The BraTS 2020 challenge dataset is a retrospective collection of 2,640 brain glioma multi-parametric magnetic resonance imaging (mpMRI) scans, from 660 patients, acquired at 23 geographically-distinct institutions under routine clinical conditions, i.e., with varying equipment and acquisition protocols [2,3] (https://wiki.cancerimagingarchive.net/pages/viewpage.action?pageId=24282666) . The exact mpMRI scans included in the BraTS 2020 challenge dataset are a) native (T1) and b)

post-contrast T1-weighted (T1Gd), c) T2-weighted (T2), and d) T2-weighted Fluid Attenuated Inversion Recovery (T2-FLAIR). Notably, the BraTS 2020 dataset was utilized in the first instance of the first ever federated learning challenge, namely the FeTS 2021 challenge (https://miccai.fets.ai/) that ran in conjunction with the MICCAI 2021 conference. Standardized pre-processing has been applied to all the BraTS mpMRI scans. This includes conversion of the DICOM files to the NIfTI file format, co-registration to the same anatomical template (SRI24) [4], resampling to a uniform isotropic resolution ($1mm^3$), and finally skull-stripping [5]. The pre-processing pipeline is publicly available through the Cancer Imaging Phenomics Toolkit (CaPTk) [4,6] and the FeTS tool [7]. The detailed pre-processing steps and annotation protocol for the BraTS dataset are available in [8]

1.1.3 Benchmark Assets

A *Data Preparation* MLCube container is generated to structure the input sample data the way the *Model* MLCube containers of this benchmark are able to ingest. The *Data Preparation* MLCube reads input data and converts it into a folder structure where each subject is defined by a folder, and checks for required inputs. For the current pilot study these inputs were the 4 structural modalities as well as the ground truth annotation. Twenty cases from the BraTS2020 validation cohort were used in this pilot experiment: 5 cases x 4 hospitals. The cases are provided along with the code (more information in the Data and Code Availability sections).

This pilot benchmark included 3 state-of-the-art segmentation models: DeepMedic [9], DeepScan [10], and nnU-Net [11]. Each model was trained on the 369 patients from the BraTS2020 training dataset, in a central fashion, and containerized to *Model* MLCube. The training data was not included in the evaluation dataset.

1.1.3.1 Benchmark Results

For each *Model* MLCube predictions were generated across 4 *Data Owners* (hereafter referenced as H1, H2, H3 and H4) from BraTS2020, 2 containing 5 cases, 1 containing 4 and the last one containing 6, for a total of 20 cases. These predictions are compared with the ground truth annotations through the *Evaluation Metrics* MLCube. In this task, metrics such as the Dice Similarity Coefficient (DSC), the 95th percentile of the Hausdorff distance, as well as Precision were measured. Each *Data Owner* was set up as a separate Google Cloud Provider (GCP) Virtual Machine instance (specifications: 16 CPU cores, 128GB of RAM and 1 NVIDIA

Tesla T4) and a MedPerf client was installed to execute the benchmarks. Aggregated DSC results for this pilot study included the Supplementary Table 1:

	Dice Similarity Coefficient		
**Testing Hospital**	**DeepMedic**	**nnU-Net**	**DeepScan**
**H1**	**0.8935**	**0.9150**	**0.9249**
**H2**	**0.9614**	**0.9703**	**0.7401**
**H3**	**0.9532**	**0.9672**	**0.9626**
**H4**	**0.8440**	**0.9570**	**0.8649**

Supplementary Table 1. Benchmarking popular segmentation models on Brain Tumor Segmentation (BraTS) 2020 data corresponding to 4 different hospitals (H1, H2, H3, and H4).

1.1.3.2 Limitations & Observations shared by Pilot Study Participants

- The BraTS data have been well-curated. This preprocessing step is an additional effort that needs to be undertaken by operators interested in executing such algorithms on their own local data. This is a major common challenge that needs to be considered carefully due to time and budget constraints.
- The BraTS preprocessing pipeline requires manual quality checks at multiple steps (i.e., after multimodality co-registration, brain extraction, and generation of automated segmentations). The current MedPerf MLCube interfaces did not provide a way to extract outputs from different steps of a computational pipeline to enable such manual quality checking, thereby relying completely on fully-automated processes.
- Higher level limitations: Use-inspired Quality Control (QC) tools are needed to contribute in the automation of data inclusion, based on their quality.

1.1.4 Pilot Study 2 - Pancreas Segmentation

1.1.4.1 Participating Institutions

- Harvard School of Public Health, Boston, USA
- Dana-Farber Cancer Institute, Boston, USA

1.1.4.2 Clinical Task

Precise organ segmentation using computed tomography (CT) images is an important step for medical image analysis and treatment planning. Pancreas segmentation involves important challenges due to the small volume and irregular shapes of the areas of interest. In this pilot study the goal was to perform federated evaluation across two different sites using MedPerf for the task of pancreas segmentation, to test the generalizability of a model trained on only one of these sites

1.1.4.3 Data Description

Two separate datasets were utilized in this pilot experiment. The first of which is the Multi-Atlas Labeling Beyond the Cranial Vault (BTCV) dataset, which is publicly available through synapse platform(https://www.synapse.org/#!Synapse:syn3193805). Abdominal CT images from 50 metastatic liver cancer patients and the postoperative ventral hernia patients were acquired at the Vanderbilt University Medical Center(https://www.synapse.org/#!Synapse:syn3193805). The abdominal CT images were registered using NiftyReg [12,13]. In addition to the BTCV dataset, another publicly available dataset from TCIA (The Cancer Imaging Archives) was utilized (https://wiki.cancerimagingarchive.net/display/Public/Pancreas-CT); the National Institute of Health Clinical Center curated this dataset with 80 abdominal scans, from 53 male and 27 female subjects. Of which 17 patients had known kidney donations that confirmed healthy abdominal regions, and the remaining patients were selected after examination confirmed that the patients had neither pancreatic lesions nor any other significant abdominal abnormalities. Visual inspection of both datasets confirmed that they were appropriate and complementary candidates for the pancreas segmentation task. Out of the 130 total cases, 10 subjects were used for inference, 5 from each dataset. Patients with IDs 1 to 5 were randomly chosen from each dataset.

1.1.5 Benchmark Assets

In this pilot study benchmark the *Data Preparation* MLCube processed CT images and saved them as numpy arrays. In general, 3D volumes of abdominal CT scans can have different spatial orientation based on the type of acquisition tool used and how it was placed. So the *Data Preparation* MLCube applied a set of preprocessing steps (i.e. rotations, scaling) to the BTCV volumes to become aligned with the TCIA volumes. The *Data Preparation* MLCube converted

labels into NIfTI data format. Since the BTCV dataset is a multi-organ dataset, its labels were altered such that all organs other than the pancreas are regarded as background.

The *Model* MLCube utilized Recurrent Saliency Transformation Network (RSTN) model [14] for pancreas segmentation in CT images whose codebase is publicly available in PyTorch (https://github.com/twni2016/OrganSegRSTN_PyTorch). The model was already pretrained on the TCIA dataset on patients subjects with IDs 21 to 82 (60 subjects), as described in their GitHub repository (https://github.com/twni2016/OrganSegRSTN_PyTorch#5-pre-trained-models-on-the-nih-dataset).
The RSTN model is a 2-stage model: in coarse stage, inference is done on each 2D slice of the three possible planar views, and in the fine stage, previous stage predictions are fused together and processed. The *Model* MLCube implemented saved all the predictions as compressed numpy arrays in a unified structure ready for evaluation.

The *Evaluation Metrics* MLCube finally evaluated the output segmentations generated from the *Model* MLCube to produce scores using the DSC metric.

1.1.5.1 Benchmark Results

To simulate the *Data Owners* hosting the TCIA and BCTV datasets, two virtual instances were setup on GCP (specifications of each: 8 CPU cores, 40GB of RAM, and 1 NVIDIA Tesla T4) each one with the MedPerf client installed. The pretrained model was evaluated using the default MedPerf server on the two virtual instances. Coarse inference DSC was recorded at 68.01% (TCIA) and 50.44% (BTCV), while the fine inference DSC resulted in 81.30% (TCIA) and 67.27% (BTCV).

1.1.5.2 Limitations & Observations shared by Pilot Study Participants

Similar to the previous pilot study observation, in real world scenarios where pancreas segmentation benchmarks are carried out, the *Benchmark Committee* must provide a preprocessing reference implementation to avoid data preparation issues that may arise due to the image orientation and scale so that input data are harmonized across all participating *Data Owners* and submitted models can be correctly evaluated. For now, it seems that the MedPerf ecosystem can be a solution for the mentioned concerns. The *Benchmark Committee* will have the authority to provide instructions for *Model* MLCube authors to prepend the necessary data

preprocessing logic to their models, and ensure the compatibility between the possibly variable models input and the unified benchmark data preparation output.

1.1.6 Pilot Study 3 - Surgical Workflow Phase Recognition

1.1.6.1 Participating institutions

- University Hospital of Strasbourg, France
- Policlinico Universitario Agostino Gemelli, Rome, Italy
- Azienda Ospedaliero-Universitaria Sant'Andrea, Rome, Italy
- Fondazione IRCCS Ca' Granda Ospedale Maggiore Policlinico, Milan, Italy
- Monaldi Hospital, Naples, Italy

1.1.6.2 Clinical Task

Surgical phase recognition is a classification task in which each video frame from a recorded surgery is assigned to a predefined phase that gives a coarse description of the surgical workflow [15]. This task is a building block for context-aware systems that help in assisting surgeons for better Operating Room (OR) safety [16].

1.1.6.3 Data description

In this pilot study data from Multichole2022 [17] was used. Multichole2022 is a multicentric dataset comprising videos of recorded laparoscopic cholecystectomy surgeries, annotated for the task of surgical phase recognition. The dataset consists of 180 videos in total, of which 56 videos were used as the evaluation dataset while the rest of the videos (i.e., 124) were used to train a centralized model. The videos were acquired from 5 hospitals: 32 videos from the University Hospital of Strasbourg, France (part of public dataset Cholec80 [18]), and 6 videos were taken from each of the following Italian hospitals: Policlinico Universitario Agostino Gemelli, Rome; Azienda Ospedaliero-Universitaria Sant'Andrea, Rome; Fondazione IRCCS Ca' Granda Ospedale Maggiore Policlinico, Milan; and Monaldi Hospital, Naples. These data is private. Videos were annotated according to the Multichole2022 annotation protocol described in [17] on 6 separate surgical phases: Preparation, Hepatocytic Triangle Dissection, Clipping and Cutting, Gallbladder Dissection, Gallbladder Packaging, and Cleaning and Coagulation.

1.1.6.4 Benchmark Assets

In this study the *Data Preparation* MLCube transformed raw data (ie. videos and labels) into a standard format to be used by the *Model* MLCube subsequently. Specifically, the *Data Preparation* MLCube extracted video frames at a rate of 1 frame per second, cropped them into 250x250 images, and associated each frame with the ground truth label.

The *Model* MLCube implemented the TeCNO model [19], which consists of two inference stages: feature extraction by a ResNet50 model, and prediction by a multi-stage temporal convolutional network model. The *Model* MLCube generated predictions for each frame of each video, which were used by the *Evaluation Metrics* MLCube for model performance evaluation. Unlike in [19] , Multichole2022 has no tool labels, and thus the model in this pilot experiment involved only phase classification training. The model was trained beforehand separately on each hospital's dataset, generating 5 models. Model weights are private for now. Unlike the other pilot studies, this benchmark used the same model architecture trained separately on data from each different hospital (i.e., *Data Owner*). Moreover, compared to the other pilot studies, this study utilized privately owned data demonstrating how MedPerf can operate in privacy-constrained environments.

Following the most common metrics used for assessing surgical phase recognition performance, the *Evaluation Metrics* MLCube contained measurement of the following metrics: Accuracy, F1-score, Precision, Recall, and Jaccard Score.

1.1.6.5 Benchmark Results

	**Testing Hospitals**				
**Training Hospital**	H1	H2	H3	H4	H5
H1	**78.2**	62.52	38.94	61.19	59.92
H2	41.98	**76.06**	44.47	27.01	42.43
H3	49.65	65.15	**63.42**	67.74	56.97
H4	41.72	50.5	40.62	**66.17**	56.88
H5	39.42	51.15	30.93	41.49	**75.94**

Supplementary Table 2**.** F1-score results for Pilot 3. In this pilot the overall F1-score metric was reported for each performed inference experiment. Rows correspond to the model used, and columns correspond to the data on which inference was run. H1 to H5 correspond to the 5 hospital sub-datasets in Multichole2022; for further details on the data acquisition, consult reference [17] from the main text.

1.1.6.6 Limitations & Observations shared by Pilot Study Participants

- Data preparation is a very important step in surgical videos. Valid datasets need to be processed accordingly, and invalid ones should be identified and rejected during the dataset registration process. The *Benchmark Committee* is the ultimate authority to make sure that the *Data Preparation* MLCube is imposed strictly across *Data Owners*. This is a necessary but not trivial task and tools that make this task easier (e.g., Quality Control) should be further investigated.
- Data annotation is also a major challenge. In particular when it comes to a benchmark an annotation protocol consensus is required. Perhaps multiple annotation protocols can be explored within a single benchmark depending on the group's requirements.

1.2 Pilot Study 4 - Cloud Experiments

We proceeded to further validate MedPerf on the cloud. Towards this end, we executed various parts of the MedPerf architecture across different cloud providers. Google Cloud Platform (GCP) was used across all experiments for hosting the MedPerf server. The Brain Tumor Segmentation (BraTS) Benchmark (Pilot 1), as well as part of the Pancreas Segmentation Benchmark (Pilot 2), were executed inside a GCP Virtual Machine with 128GB of RAM and an Nvidia T4. The Surgical Workflow Phase Recognition Benchmark (Pilot 3) was executed on internal servers due to privacy concerns.

Lastly, we ran a Chest X-Ray Pathology Classification Benchmark to demonstrate the feasibility of running federated evaluation across different cloud providers. We used the CheXpert dataset (https://stanfordmlgroup.github.io/competitions/chexpert/) and a Densenet pre-trained model from the TorchXRayVision library (https://github.com/mlmed/torchxrayvision). For this, the CheXpert [20] small validation dataset was partitioned into 4 splits, and executed inside Virtual

Machines provisioned within AWS, Alibaba, Azure, and IBM. As with the other pilot experiments, all results were uploaded to the MedPerf server hosted on GCP.

Supplementary Figure 1 summarizes which cloud provider each MedPerf component (i.e., server, client) and dataset was executed on, and in the bottom part we present where the data was collected for all pilots. The code for this pilot is available inside MedPerf's repository (https://github.com/mlcommons/medperf#pilot-4---cloud-experiments)

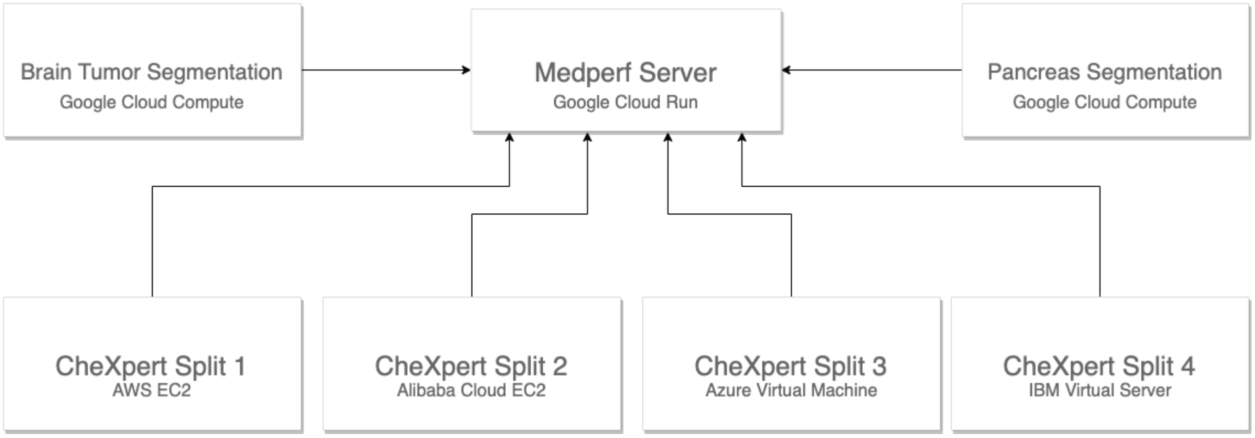

Supplementary Figure 1. Execution of pilot benchmarks on multiple cloud providers. Details are provided in our repository: https://github.com/mlcommons/MedPerf.

2 Consortia

2.1 AI4SafeChole Consortium

Giovanni Guglielmo Laracca, Ludovica Guerriero, Andrea Spota, Claudio Fiorillo, Giuseppe Quero, Segio Alfieri, Ludovica Baldari, Elisa Cassinotti, Luigi Boni, Diego Cuccurullo, Guido Costamagna, Bernard Dallemagne

2.2 BraTS-2020 Consortium

Bjoern Menze, Christos Davatzikos, Jayashree Kalpathy-Cramer, Keyvan Farahani, John B. Freymann, Justin S. Kirby, Rivka R. Colen, Aikaterini Kotrotsou, Daniel Marcus, Mikhail Milchenko, Arash Nazeri, Hassan Fathallah-Shaykh, Roland Wiest, Andras Jakab, Marc-Andre Weber, Abhishek Mahajan, Prashant Shah, Russell Takeshi Shinohara, Chiharu Sako, Parth Sharma, Martin Rozycki, Christoph Berger

2.3 CHAOS Consortium

A. Emre Kavur, N. Sinem Gezer, Mustafa Barış, Sinem Aslan, Pierre-Henri Conze, Vladimir Groza, Duc Duy Pham, Soumick Chatterjee, Philipp Ernst, Savaş Özkan, Bora Baydar, Dmitry Lachinov, Shuo Han, Josef Pauli, Fabian Isensee, Matthias Perkonigg, Rachana Sathish, Ronnie Rajan, Debdoot Sheet, Gurbandurdy Dovletov, Oliver Speck, Andreas Nürnberger, Klaus H. Maier-Hein, Gözde Bozdağı Akar, Gözde Ünal, Oğuz Dicle, M. Alper Selver.

2.4 FeTS Consortium

Christos Davatzikos, Chiharu Sako, Michel Bilello, Satyam Ghodasara, Suyash Mohan, Evan Calabrese, Jeffrey Rudie, Javier Villanueva-Meyer, Soonmee Cha, Madhura Ingalhalikar, Manali Jadhav, Umang Pandey, Jitender Saini, Minh-Son To, Sargam Bhardwaj, Chee Chong, Marc Agzarian, Michal Kozubek, Filip Lux, Jan Michálek, Petr Matula, Miloš Keřkovský, Tereza Kopřivová, Marek Dostál, Václav Vybíhal, Joseph A. Maldjian, Chandan Ganesh Bangalore Yogananda, Marco C. Pinho, Divya Reddy, James Holcomb, Benjamin C. Wagner, Benedikt Wiestler, Bjoern Menze, Florian Kofler, Ivan Ezhov, Marie Metz, Yuriy Gusev, Krithika Bhuvaneshwar, Anousheh Sayah, Camelia Bencheqroun, Anas Belouali, Subha Madhavan, Aly Abayazeed, Kenneth Kolodziej, Michael Hill, Ahmed Abbassy, Shady Gamal, Mohamed Qayati, Mahmoud Mekhaimar, Mauricio Reyes, Rivka R. Colen, Aikaterini Kotrotsou, Philipp Vollmuth, Gianluca Brugnara, Chandrakanth J. Preetha, Felix Sahm, Klaus Maier-Hein, Maximilian Zenk, Martin Bendszus, Wolfgang Wick, Abhishek Mahajan, Ujjwal Baid, Carmen Balana Quintero, Jaume Capellades, Josep Puig, Yoon Seong Choi, Seung-Koo Lee, Jong Hee Chang, Sung Soo Ahn, Hassan F. Shaykh, Alejandro Herrera-Trujillo, Maria Trujillo, William Escobar, Ana Abello, Jose Bernal, Jhon Gómez, Pamela LaMontagne, Daniel Marcus, Ashok Srinivasan, J. Rajiv Bapuraj, Arvind Rao, Nicholas Wang, Ota Yoshiaki, Toshio Moritani, Sevcan Turk, Joonsang Lee, Snehal Prabhudesai, Bjoern Menze, Hongwei Li , Tobias Weiss, Michael Weller, Andrea Bink, Bertrand Pouymayou, Florian Kofler, Alexandre Xavier Falcão, Samuel B. Martins, Bernardo C. A. Teixeira, Flávia Sprenger, David Menotti, Diego R. Lucio, Simone P. Niclou, Olivier Keunen, Ann-Christin Hau, Enrique Pelaez, Heydy Franco-Maldonado, Francis Loayza, Sebastian Quevedo, Johannes Trenkler, Josef Pichler, Georg Necker, Andreas Haunschmidt, Stephan Meckel, Pamela Guevara, Esteban Torche, Cristobal Mendoza, Franco Vera, Elvis Ríos, Eduardo López, Sergio A. Velastin, Martin Vallières, David Fortin, Martin Lepage, Fanny Morón, Jacob Mandel, Gaurav Shukla , Spencer Liem , Gregory S. Alexandre , Joseph Lombardo , Joshua D. Palmer , Adam E. Flanders , Adam P. Dicker , Godwin Ogbole, Dotun Oyekunle, Olubunmi Odafe-Oyibotha, Babatunde Osobu, Mustapha Shu'aibu, Mayowa Soneye, Farouk Dako, Adeleye Dorcas, Derrick Murcia, Eric Fu, Rourke Haas, John Thompson, David Ryan Ormond, Stuart Currie, Kavi Fatania, Russell Frood, Amber L. Simpson, Jacob J. Peoples, Ricky Hu, Danielle Cutler, Fabio Y. Moraes, Anh Tran, Mohammad Hamghalam

3 Supplementary References

1. Pati, S. *et al.* The Federated Tumor Segmentation (FeTS) Challenge. *arXiv* (2021).
2. Menze, B. H. *et al.* The multimodal brain tumor image segmentation benchmark (BRATS). *IEEE Trans. Med. Imaging* **34**, 1993–2024 (2015).
3. Bakas, S. *et al.* Advancing The Cancer Genome Atlas glioma MRI collections with expert segmentation labels and radiomic features. *Sci. Data* **4**, 170117 (2017).
4. Rohlfing, T., Zahr, N. M., Sullivan, E. V. & Pfefferbaum, A. The SRI24 multichannel atlas of normal adult human brain structure. *Hum. Brain Mapp.* **31**, 798–819 (2010).
5. Baid, U. *et al.* Nimg-32. the federated tumor segmentation (fets) initiative: the first real-world large-scale data-private collaboration focusing on neuro-oncology. *Neuro Oncol.* **23**, vi135–vi136 (2021).
6. Pati, S. *et al.* The cancer imaging phenomics toolkit (captk): technical overview. *Brainlesion* **11993**, 380–394 (2020).
7. Pati, S. *et al.* The federated tumor segmentation (FeTS) tool: an open-source solution to further solid tumor research. *Phys. Med. Biol.* (2022) doi:10.1088/1361-6560/ac9449.
8. Bakas, S. *et al.* Identifying the Best Machine Learning Algorithms for Brain Tumor Segmentation, Progression Assessment, and Overall Survival Prediction in the BRATS Challenge. *arXiv* (2018).
9. Kamnitsas, K. *et al.* Efficient multi-scale 3D CNN with fully connected CRF for accurate brain lesion segmentation. *Med. Image Anal.* **36**, 61–78 (2017).
10. McKinley, R., Meier, R. & Wiest, R. Ensembles of Densely-Connected CNNs with Label-Uncertainty for Brain Tumor Segmentation. in *Brainlesion: Glioma, Multiple Sclerosis, Stroke and Traumatic Brain Injuries: 4th International Workshop, BrainLes 2018, Held in Conjunction with MICCAI 2018, Granada, Spain, September 16, 2018, Revised Selected Papers, Part II* (eds. Crimi, A. et al.) vol. 11384 456–465 (Springer International Publishing, 2019).
11. Isensee, F., Jaeger, P. F., Kohl, S. A. A., Petersen, J. & Maier-Hein, K. H. nnU-Net: a self-configuring method for deep learning-based biomedical image segmentation. *Nat. Methods* **18**, 203–211 (2021).
12. Modat, M. *et al.* Global image registration using a symmetric block-matching approach. *J Med Imaging (Bellingham)* **1**, 024003 (2014).
13. Modat, M. *et al.* Fast free-form deformation using graphics processing units. *Comput. Methods Programs Biomed.* **98**, 278–284 (2010).
14. Yu, Q. *et al.* Recurrent Saliency Transformation Network: Incorporating Multi-Stage Visual Cues for Small Organ Segmentation. *arXiv* (2017) doi:10.48550/arxiv.1709.04518.
15. Padoy, N. *et al.* Statistical modeling and recognition of surgical workflow. *Med. Image Anal.* **16**, 632–641 (2012).
16. Mascagni, P. *et al.* A computer vision platform to automatically locate critical events in surgical videos: documenting safety in laparoscopic cholecystectomy. *Ann. Surg.* **274**, e93–e95 (2021).
17. Kassem, H., Alapatt, D., Mascagni, P., Karargyris, A. & Padoy, N. Federated Cycling (FedCy): Semi-supervised Federated Learning of Surgical Phases. *IEEE Trans. Med. Imaging* **PP**, (2022).
18. Twinanda, A. P. *et al.* Endonet: A deep architecture for recognition tasks on laparoscopic videos. *IEEE Trans. Med. Imaging* **36**, 86–97 (2017).
19. Czempiel, T. *et al.* TeCNO: Surgical Phase Recognition with Multi-stage Temporal Convolutional Networks. in *Medical image computing and*

computer assisted intervention – MICCAI 2020: 23rd international conference, lima, peru, october 4–8, 2020, proceedings, part III (eds. Martel, A. L. et al.) vol. 12263 343–352 (Springer International Publishing, 2020).

20. Irvin, J. *et al*. CheXpert: A Large Chest Radiograph Dataset with Uncertainty Labels and Expert Comparison. *AAAI* **33**, 590–597 (2019).
